# How Musculoskeletal Tumor Management Changed During the COVID-19 Pandemic: Data from a Nationwide Questionnaire Survey of Hospitals Specializing in Musculoskeletal Tumors in Japan

**DOI:** 10.3390/curroncol32080453

**Published:** 2025-08-12

**Authors:** Takeshi Morii, Shintaro Iwata, Kensaku Yamaga, Masanori Okamoto, Kosei Ando, Takaaki Tanaka, Jun Nishida

**Affiliations:** 1 Department of Orthopaedic Surgery, School of Medicine, Kyorin University, 6-20-2 Shinkawa, Mitaka-shi 181-8611, Tokyo, Japan; 2 Department of Musculoskeletal Oncology and Rehabilitation Medicine, National Cancer Center Hospital, 5-1-1 Tsukiji, Chuo-ku 104-0045, Tokyo, Japan; shiwata@ncc.go.jp; 3 Department of Orthopedic Surgery, Faculty of Medicine, Tottori University, 36-1 Nishi-cho, Yonago 683-8504, Tottori, Japan; kyamaga@tottori-u.ac.jp; 4 Department of Orthopaedic Surgery, Shinshu University School of Medicine, 3-1-1 Asahi, Matsumoto 390-8621, Nagano, Japan; ryouyuma@shinshu-u.ac.jp; 5 Department of Orthopaedic Surgery, Shiga University of Medical Science, Seta Tsukinowa-cho, Otsu 520-2192, Shiga, Japan; kosei@belle.shiga-med.ac.jp; 6 Department of Orthopedics and Rehabilitation Medicine, Unit of Surgery, Division of Medicine, Faculty of Medical Sciences, University of Fukui, 23-3 Shimoaizuki, Matsuoka 910-1193, Fukui, Japan; takaaki.tanaka.77@gmail.com; 7 Department of Orthopedic Surgery, Tokyo Medical University, 6-7-1 Nishishinjuku, Shinjuku-ku 160-0023, Tokyo, Japan; jnishida@f2.dion.ne.jp

**Keywords:** COVID-19, nationwide survey, malignant bone tissue tumor, malignant soft tissue tumor

## Abstract

Background: While changes in clinical practice during the COVID-19 pandemic in Japan have been widely studied, data specific to bone and soft tissue tumor care remain limited. Methods: A nationwide web-based survey was conducted among hospitals specializing in musculoskeletal tumors. It assessed the occurrence of COVID-19-related events (patient infections, outbreak clusters, and staff infections), delays in referral and diagnosis, postponement or cancellation of specific treatments, and changes in institutional management strategies. Results: Seventy-eight hospitals (91.7% of all specialized centers) responded. Patient infections, outbreak clusters, and staff infections were reported by 28.2%, 48.7%, and 53.8% of hospitals, respectively. While radiological exams and biopsies were largely maintained, patient referrals decreased significantly. Surgical treatment was more affected than chemotherapy or radiotherapy. Strategy changes included surgery delays or cancellations (48.7%) and prolonged follow-up intervals (20.5%). Among COVID-19-related factors, only direct patient infections were significantly associated with institutional changes in treatment policy. Conclusions: The pandemic substantially disrupted outpatient services and surgical care in musculoskeletal oncology. Patient infection was the main driver of treatment strategy modifications.

## Simple Summary

The COVID-19 pandemic had a significant impact on cancer care, but its effects on bone and soft tissue tumor treatment in Japan were not well known. We conducted a nationwide survey of hospitals that specialize in musculoskeletal tumors to understand how their practices changed during the pandemic. While imaging and biopsy procedures were mostly maintained, fewer patient referrals and delays or cancellations in surgery were observed. The main reason for changing the treatment policy was when patients themselves became infected with COVID-19. These findings highlight how the pandemic disrupted cancer care and the importance of preparing better systems for future health crises.

## 1. Introduction

The COVID-19 pandemic resulted in devastating changes to social infrastructure. The greatest change was international and regional transfer restrictions to prevent the spread of COVID-19, resulting in limited patient access to hospitals. The increased demand for COVID-19 care, along with hospital lockdowns, restricted access, and staff infections, led to a redistribution of medical resources [1,2,3,4,5,6,7,8].

The specific characteristics of bone/soft tissue tumors are the broad range of malignancies and management modalities, including surgical intervention, perioperative chemotherapy/radiotherapy, and terminal care. In addition, the scarcity of specialized institutions and specialists should be noted. Therefore, triage based on tumor grade was proposed in the guidelines for bone and soft tissue tumors during the COVID-19 pandemic [9,10,11,12,13]. Malignant tumors were given the highest priority among orthopedic surgeries from the outset [8]. For diagnosis, lesions suspected of malignancy, pathological fractures, conditions with suspicious malignant transformations, local recurrence, and metastasis were given the highest priority [12]. For surgery, high-grade bone sarcoma, such as osteosarcoma or Ewing sarcoma; sarcoma completely cured only by surgery, such as chondrosarcoma; and high-grade soft tissue sarcoma were given higher priority [9,11,12]. High-grade primary malignant bone sarcoma should be given the highest priority for neoadjuvant/adjuvant chemotherapy [10,12]. In contrast, low-grade sarcoma or intermediate soft tissue tumors were recommended to be given lower priority for treatment, and sometimes, a wait-and-see strategy was proposed [9].

Based on the context of the recommendations, changes in the management of bone and soft tissue tumors could be anticipated. These changes may include delays in diagnosis; postponement or cancellation of surgical intervention, chemotherapy, and radiotherapy; extension of follow-up periods in outpatient clinics; and modifications in terminal care [1,2,3,4,5,13]. While several studies have reported that such quality deterioration did not affect the treatment outcomes [4,14], many have suggested a direct effect on morbidities and oncological outcomes. For example, Fitzgerald et al. showed that the morbidities caused by delays in surgical intervention for musculoskeletal tumors included prolonged pain/disability, unplanned preoperative adjuvant therapy, local tumor progression, increased systemic disease, missed surgical opportunities due to disease progression or loss to follow-up, and delays in diagnosis [2].

The status of the actual resource limitations in medical resources caused by the COVID-19 pandemic should be interpreted not only for historical records but also to establish strategies for likely future pandemics. Due to likely regional variations in sociological characteristics, COVID-19 pandemic status, and management strategies for COVID-19, specific studies in each area/country are needed. While many studies on changes in medical conditions during the COVID-19 pandemic in Japan have been published, limited information is available on clinical practices for bone and soft tissue tumors [5,6,7,8,15,16]. Therefore, in this study, we examine the changes in management quality and treatment strategy for malignant bone and soft tissue tumors in specialized hospitals in Japan during the COVID-19 pandemic via a nationwide questionnaire survey.

## 2. Materials and Methods

### 2.1. Data Collection

This study conducted a nationwide questionnaire survey of hospitals specializing in musculoskeletal tumors in Japan using a web-based questionnaire. It focused on the 85 referral hospitals authorized by the Japanese Orthopedic Association (JOA). In the context of the JOA, referral hospitals are those registered in its bone and soft tissue tumor registry. Invitations were distributed to these hospitals by the Secretariat of the 56th Annual Musculoskeletal Tumor Meeting of the JOA in 2023. The survey was conducted as part of a symposium organized by the meeting. The department chief responsible for bone and soft tissue tumor care was asked to complete the questionnaire. The questionnaire included questions about hospital characteristics (Q1) and experiences with COVID-19-related events (Q2–Q4), such as patient infections, outbreak clusters, and staff infections. It also addressed changes in treatment and management strategies for malignant musculoskeletal tumors during the COVID-19 pandemic (Q5–Q11) from April 2020 to the end of 2021 (Table 1).

Free narrative comments were allowed on each topic. Narrative descriptions that provided insight into hospital conditions during the COVID-19 pandemic were identified and presented. In this study, only the data regarding the number of treatments and the management strategy were collected. The data were collected independently of patient demographics and personal information. Therefore, ethics approval was not required for this study.

### 2.2. Data Presentation and Statistics

Descriptive statistics were calculated, and the effects of hospital type (university hospitals, local referral hospitals, or cancer centers) and COVID-19-related events on treatment quality and management strategy were analyzed. In addition, we compared the incidence of deterioration across different tumor management stages—diagnosis, treatment for primary tumors, and treatment for metastatic tumors—to identify which was most affected. In this context, “deterioration” is defined as either a decrease in the frequency of interventions or a delay in the timing of interventions. Statistical analyses were performed using chi-square tests in JMP software (version 13.0.0; SAS Institute Inc., Cary, NC, USA).

## 3. Results

### 3.1. Demographic Data

Seventy-eight hospitals responded, representing 91.7% of hospitals specializing in musculoskeletal tumors in Japan. They comprised 51 (65.3%) university hospitals, 14 (17.9%) local referral hospitals, and 13 (16.7%) cancer centers.

Among them, 22 hospitals (28.2%) reported COVID-19 infections in patients, 38 (48.7%) reported outbreak clusters, and 42 (53.8%) reported infections among musculoskeletal tumor team staff. The frequency of patient infections (*p* = 0.29), outbreak clusters (*p* = 0.23), and staff infections (*p* = 0.40) did not differ significantly among university hospitals, local referral hospitals, and cancer centers.

Narrative comments on outbreak clusters were obtained from 33 hospitals. Temporary cessation of the acceptance of referral patients and ward closure were reported by 16 (48.5%) and 5 (14.2%) hospitals, respectively. Under such conditions, the transfer of patients needing immediate management for musculoskeletal tumors was reported by two hospitals, suggesting practical management flexibility during the COVID-19 pandemic.

### 3.2. Impact on the Diagnostic Process

The impact of the COVID-19 pandemic on the diagnostic process was assessed by examining changes in patient referrals, institutional restrictions on radiological examinations (computed tomography [CT] and magnetic resonance imaging [MRI]), and the prolongation of the interval between the first visit and the performance of a biopsy (Q5–Q7). As shown in Table 2, radiological examination and biopsy procedures were maintained in most hospitals, while the number of patient referrals decreased in 29.5% of the hospitals.

**Figure 1:**
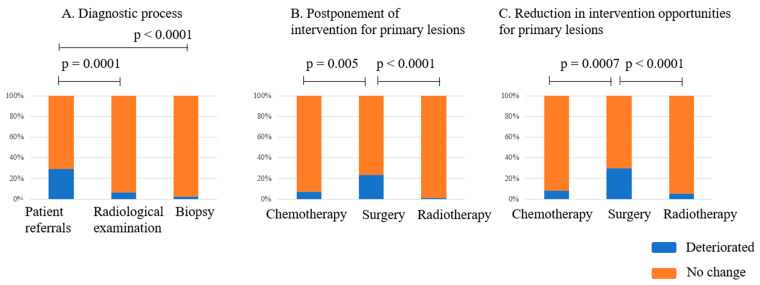
The incidence of deterioration in diagnostic and treatment processes in hospitals specializing in musculoskeletal tumors. The p-values reflect the results of post hoc tests. (**A**) The deterioration in diagnostic processes was significantly greater for patient referrals than for radiological examination and biopsy (*p* < 0.0001). (**B**) The incidence of postponing interventions for primary musculoskeletal tumors was significantly greater for surgery than for chemotherapy or radiotherapy (*p* < 0.0001). (**C**) The incidence of reductions in treatment opportunities for primary musculoskeletal tumors was significantly greater for surgery than for chemotherapy or radiotherapy (*p* < 0.0001).

Differences in deterioration rates across diagnostic components—including reduced patient referrals, restricted access to radiological examinations, and delayed biopsies—were analyzed. The overall difference was statistically significant (*p* < 0.0001), and post hoc tests showed that patient referrals experienced significantly greater deterioration than radiological examinations or biopsies (Figure 1A).

Patient referrals (*p* = 0.61), restrictions on radiological examinations (*p* = 0.20), and biopsies (*p* = 0.43) did not differ significantly by hospital type. Experiencing COVID-19 events did not affect the diagnosis process, including radiological examinations (infection of patients: *p* = 0.12; outbreak clusters: *p* = 0.60; and infections of staff: *p* = 0.77) and biopsies (infection of patients: *p* = 0.24; outbreak clusters: *p* = 0.97; and infections of staff: *p* = 0.91).

In narrative comments, a case with severe progression of the primary disease because of the self-judgment to postpone visiting the referral hospital during the COVID-19 pandemic was reported, suggesting a strong need for enlightenment of patients as to the emerging need for early intervention in high-grade sarcomas, even during a pandemic. In addition, two cases that required a differential diagnosis between postoperative surgical site infection and infection with COVID-19 were noted.

### 3.3. Impact on the Treatment Quality of Primary Malignant Musculoskeletal Tumors

The impact of the COVID-19 pandemic on the status of the treatment process, such as postponing and reducing treatment procedures for primary musculoskeletal tumors, is shown in Table 3. The impact was significantly greater for surgery than for chemotherapy and radiotherapy, both in terms of postponement and reduction in the frequency of the modalities (Figure 1B,C).

Hospital type had no significant impact on the postponement of chemotherapy (*p* = 0.41), surgery (*p* = 0.35), or radiotherapy (*p* = 0.66), nor on the reduction in chemotherapy (*p* = 0.07), surgery (*p* = 0.68), or radiotherapy (*p* = 0.50).

Experiencing COVID-19-related events did not significantly affect the postponement of interventions, including chemotherapy (patient infection: *p* = 0.14; outbreak clusters: *p* = 0.16; infections of staff: *p* = 0.41), surgery (patient infection: *p* = 0.93; outbreak clusters: *p* = 0.25; infections of staff: *p* = 0.06), or radiotherapy (patient infection: *p* = 0.41; outbreak clusters: *p* = 0.24; infections of staff: *p* = 0.19).

Similarly, COVID-19-related events did not significantly affect the reduction in interventions, including chemotherapy (patient infection: *p* = 0.83; outbreak clusters: *p* = 0.41; infections of staff: *p* = 0.21), surgery (patient infection: *p* = 0.18; outbreak clusters: *p* = 0.75; infections of staff: *p* = 0.78), or radiotherapy (patient infection: *p* = 0.09; outbreak clusters: *p* = 0.97; infections of staff: *p* = 0.19).

In the narrative comments, two cases were noted in which systemic dissemination occurred during the postponement period of surgical intervention, suggesting the probability of a relationship between worse prognosis and COVID-19 infection. The need for mental health care, especially for pediatric patients undergoing long-term systemic chemotherapy, was reported by two hospitals.

### 3.4. Changes in the Management Status of Metastatic Bone Tumors

**Figure 2:**
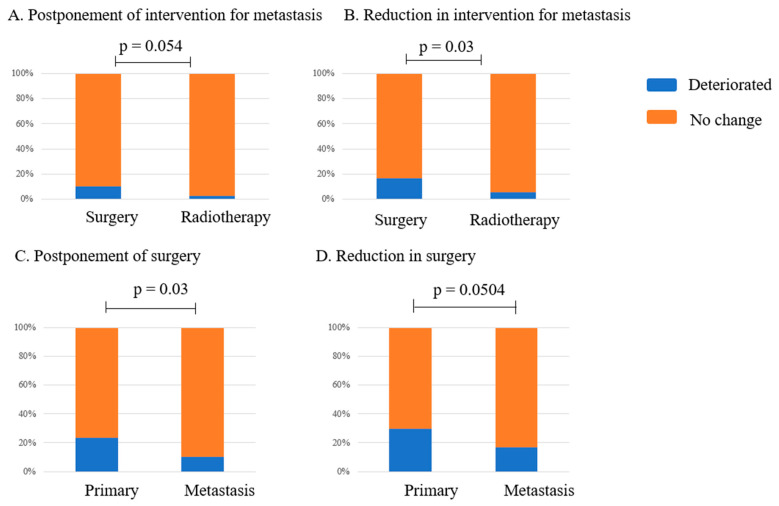
The incidence of deterioration in radiotherapy and surgery for metastatic bone tumors. (**A**) The incidence of postponing interventions for metastasis. (**B**) The incidence of reducing treatment opportunities for metastasis. (**C**) The incidence of postponing surgery for primary and metastatic lesions. (**D**) The incidence of reductions in surgery for primary and metastatic lesions.

The impact of the COVID-19 pandemic on the treatment of metastatic bone tumors, such as postponement and reduction in treatments for metastatic bone tumors, is shown in Table 4. The incidence of treatment delays did not differ significantly between surgery and radiotherapy, whereas the reduction in application frequency was significantly greater for surgery than for radiotherapy (Figure 2A,B). The type of hospital did not significantly affect the postponement of surgery (*p* = 0.09) or radiotherapy (*p* = 0.46), nor did it affect the reduction in surgery (*p* = 0.47) or radiotherapy (*p* = 0.20). Infections in patients did not significantly affect the postponement of surgery (*p* = 0.54) or radiotherapy (*p* = 0.49), nor did they affect the reduction in surgery (*p* = 0.64) or radiotherapy (*p* = 0.91). Outbreak clusters did not significantly affect the postponement of surgery (*p* = 0.50) or radiotherapy (*p* = 0.09), nor did they affect the reduction in surgery (*p* = 0.83) or radiotherapy (*p* = 0.28). Infections among musculoskeletal team staff did not significantly affect the postponement of surgery (*p* = 0.32) or radiotherapy (*p* = 0.07), nor did they affect the reduction in surgery (*p* = 0.54) or radiotherapy (*p* = 0.84).

Surgical delays were more commonly observed in cases of primary musculoskeletal tumors compared to metastatic bone tumors (Figure 2C), whereas the difference in the reduction in surgical procedures was not significant (Figure 2D). The frequency of deterioration in radiotherapy—measured by postponement (*p* = 0.56) and reduction in intervention (*p* = 0.97)—did not differ significantly between primary and metastatic bone tumors.

### 3.5. Change in the Quality of Palliative Care

The impact of COVID-19 on the quality of palliative care was described. During the pandemic, 16 hospitals (20.5%) reported a decline in quality, while 57 hospitals (73.0%) indicated that quality remained unchanged. The remaining five hospitals did not provide palliative care. In the narrative comments, two hospitals noted an increase in home care. The type of hospital (*p* = 0.28), infections in patients (*p* = 0.18), outbreak clusters (*p* = 0.95), and infections among musculoskeletal team staff (*p* = 0.91) did not significantly affect the contents of palliative care.

### 3.6. Impact on Treatment Strategies

**Figure 3:**
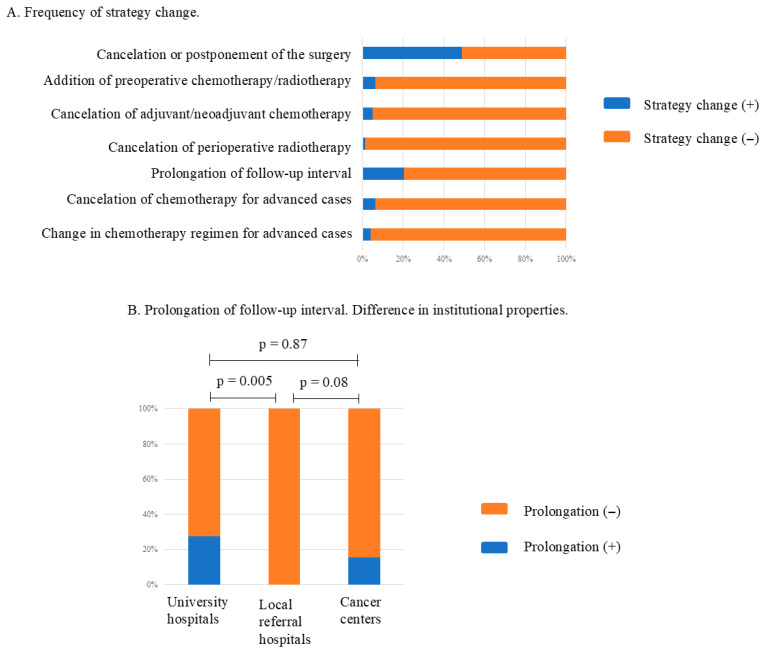
(**A**) The incidence of changes in institutional strategies for managing musculoskeletal tumors during the COVID-19 pandemic. (**B**) Differences in the incidence of prolongation of follow-up periods among university hospitals, local referral hospitals, and cancer centers.

Finally, changes in the treatment strategy for malignant bone/soft tissue tumors during the COVID-19 pandemic were assessed. A total of 64.1% of hospitals reported some deviations from their usual practices. The cancellation/postponement of surgery that is usually performed as an initial treatment (48.7%) and the extension of follow-up intervals (20.5%) were evident (Figure 3A). Prolongation of follow-up intervals was significantly more common in the university and cancer center hospitals than in the local referral hospitals (*p* = 0.02, Figure 3B). The post hoc test revealed that the frequency of prolongation was significantly greater in the university hospitals than in the local referral hospitals. The type of hospital did not significantly affect any of the following treatment decisions: cancellation or postponement of surgery (*p* = 0.40), addition of preoperative chemotherapy or radiotherapy (*p* = 0.36), cancellation of adjuvant or neoadjuvant chemotherapy (*p* = 0.43), cancellation of perioperative radiotherapy (*p* = 0.65), cancellation of chemotherapy for advanced cases (*p* = 0.39), or changes in chemotherapy regimens for advanced cases (*p* = 0.51).

**Figure 4:**
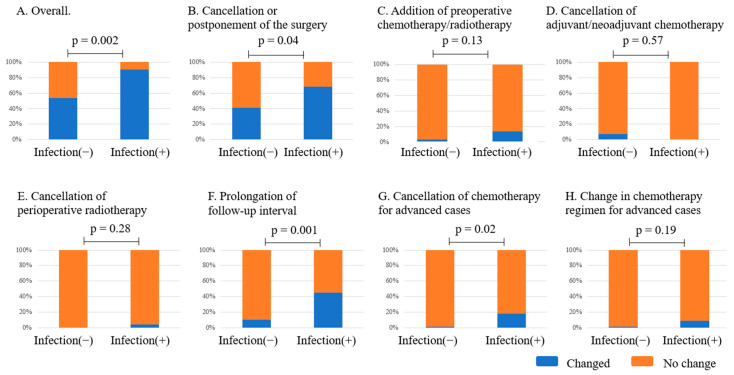
The impact of COVID-19 infections in patients with musculoskeletal tumors on the institutional strategy for managing musculoskeletal tumors.

Hospitals that experienced COVID-19 infections in patients tended to have more occasions of changes to treatment strategies for malignant bone/soft tissue sarcoma in several aspects (Figure 4). For example, 90% of the hospitals that experienced COVID-19 infections in patients needed to change the treatment strategy, compared to only 53.6% of hospitals that did not (Figure 4A). In particular, cancellation or postponement of surgery (Figure 4B), prolongation of the follow-up interval (Figure 4F), and cancellation of chemotherapy for advanced cases (Figure 4G) were significantly evident. In contrast, outbreak clusters (*p* = 0.86) and COVID-19 infections among staff (*p* = 0.14) were not associated with changes in treatment strategy.

The narrative comments from two hospitals declared they maintained the standard strategy for managing primary highly malignant sarcoma.

## 4. Discussion

### 4.1. Institutional Function

This study demonstrated that the COVID-19 pandemic had a significant impact on outpatient services, surgical treatment, and palliative care. In contrast, radiological and pathological diagnostic procedures, as well as radiotherapy and chemotherapy, were largely maintained. Among the COVID-19-related events that occurred in the hospitals, infections in patients rather than cluster outbreaks or infections in department staff significantly impacted the treatment strategy.

#### 4.1.1. Outpatients’ Clinic and Diagnosis

Several guidelines recommended reducing outpatient visits based on patients’ status [12]. Telemedicine was also recommended during the COVID-19 pandemic [17]. Only patients with suspected malignant or locally aggressive benign lesions should be considered for thorough diagnostic evaluation. In addition, follow-up should be restricted to patients with postoperative unstable conditions or signs of relapse in the field of orthopedic oncology [12]. Indeed, decreases in new registrations or increases in the use of telemedicine were reported in musculoskeletal oncology care units during the COVID-19 pandemic [18,19].

Regarding outpatient clinic management, this study noted significant reductions in patient referrals (Figure 1A), likely due to triage by local referral source clinics, limitations in transfers in the area, and patients choosing not to be referred to specialist hospitals. Indeed, a case of self-restriction of referral due to pandemic conditions was reported in the narrative comments, unfortunately resulting in disease progression. Significant delays in diagnosis during the COVID-19 pandemic were reported in Spain [4]. However, in our cohort, diagnostic delays were not significant (Table 2), suggesting the maintenance of diagnostic functions in hospitals in Japan.

This study observed prolonged follow-up intervals in 20% of hospitals (Figure 3A). Because the specialists could discriminate between high-risk and low-risk cases, it can be easily speculated that follow-up times were triaged. Notably, hospitals that experienced COVID-19 infections among patients were more likely to extend follow-up intervals (Figure 4F). The following reasons are presumed to explain this trend. First, hospitals that experienced COVID-19 cases may have extended follow-up periods as a precautionary measure to monitor for delayed complications or recurrence. Second, reduced clinic capacity and scheduling delays due to infection control measures could have unintentionally prolonged follow-up. Third, greater caution toward high-risk patients may have led to intentionally longer observation periods.

#### 4.1.2. Surgical Intervention

A significant decrease in the total number of orthopedic surgeries in various fields in Japan, especially in the early period of the COVID-19 pandemic, has been reported based on the National Database of Health Insurance Claims and Specific Health Checkups of Japan (NDB) [5] and a nationwide hospital survey [15]. Importantly, compared to surgery for benign tumors, a decrease in surgery for malignant tumors was evident in the NDB data [5]. Delays in surgical interventions for primary musculoskeletal tumors were reported in diverse areas worldwide [2,3,4]. In our study, we observed significant delays and a reduced frequency of surgical interventions for primary musculoskeletal tumors. Additionally, the frequency of changes in surgical strategy increased, suggesting that surgical treatment—particularly for primary lesions—was the most affected modality among clinical practices during the pandemic (Figure 1B,C and Figure 3A). A study by COVIDSurg Collaborative showed that COVID-19 infection in patients was a direct reason for postponing or cancelling the planned cancer surgery in only 1.1% of cases [1]. While the actual number of infected patients was small, an exaggerated impact on the decision-making for surgery may be expected. Nonetheless, we could not identify other factors related to postponing surgery in the COVIDSurg Collaborative data, although the status of the lockdown, the financial status of the nation, and patients’ Eastern Cooperative Oncology Group status should be considered risk factors for cancelling surgery during a pandemic [1]. Notably, this study suggests that among the types of cancer, cases diagnosed with sarcoma were at risk of cancellation.

Metastatic bone tumors sometimes present emerging conditions, such as an impending fracture or spinal palsy, which might be given higher priority for management during a pandemic [9,10,20]. In this study, the incidence of postponement of surgery was smaller for metastatic than primary tumors, although the reduction in application frequency did not differ significantly, suggesting that, in some cases, emergency surgical intervention was more frequent in metastasis (Figure 2C,D). Clinical practice guidelines for the management of bone metastases have been published, and many referral hospitals follow these guidelines [21]. Therefore, it is presumed that there is little variation among institutions regarding the indication for surgery in cases with oncologic urgency.

#### 4.1.3. Chemotherapy and Radiotherapy

Many guidelines recommended triaging the application of chemotherapy during the COVID-19 pandemic. Most recommended prioritizing indispensable neoadjuvant/adjuvant chemotherapy for osteosarcoma and Ewing sarcoma [9,10,12]. Regarding radiotherapy, immediate application is recommended for cases with a strong need, such as those with rapid tumor progression [12].

This study suggests that, compared to surgery, chemotherapy and radiotherapy services were largely maintained during the pandemic (Figure 1B,C, Figure 3A, and Figure 4D,E). A study in Spain reported no significant differences in neoadjuvant/adjuvant chemotherapy and neoadjuvant/adjuvant palliative chemotherapy between controls and a cohort with COVID-19, supporting our results [4]. While institutions placed restrictions on accessing both the radiotherapy department and the operating room, other factors, such as a lack of intensive care unit space, a lack of resources for anesthesia, and limitations in anesthesia for patients with COVID-19, which affect only the performance of surgical interventions, had less of an impact on radiotherapy performance.

Compared to surgery, radiotherapy performance for metastasis was less affected both in frequency and timing (Figure 2A,B). Radiotherapy was expected to be applied for cases with a strong need for an emerging intervention for metastasis instead of surgical intervention. Our results suggest an adequate maintenance of radiotherapy services even during the COVID-19 pandemic [12].

#### 4.1.4. Terminal Care

In this study, several hospitals reported a decline in the quality of palliative care. In some institutions with confirmed COVID-19 cases, palliative chemotherapy may have been cancelled; however, changes in treatment strategies for advanced-stage patients were relatively uncommon (Figure 3A). This result is supported by a study that suggested no significant difference in neoadjuvant/adjuvant palliative chemotherapy between controls and a cohort with COVID-19 [4]. Nevertheless, in the narrative descriptions, several hospitals reported access restrictions for patients and their families. While palliative care was considerably maintained at hospitals in Japan, social factors such as regional access limitations might have contributed to inconveniences in accessing palliative care services.

### 4.2. The Effect of COVID-19-Related Events

Possible causes of changes in hospital function include sociological factors (e.g., restrictions on transfers, lockdowns, or recommendations in guidelines), institutional factors (e.g., a lack of medical resources and institutional pandemic responses or lockdown), pathophysiological factors (e.g., infections in patients or staff and risk management in patients/staff in close contact with those with COVID-19), or psychological factors (e.g., patients’ decisions or institutional policy changes due to the pandemic) [1].

This study focused on COVID-19-related events such as infections in patients, outbreak clusters in the hospital, or infections in musculoskeletal tumor team staff in the department under the hypothesis that these factors could impact changes in hospital function or treatment policy. One criticism might be that the question should consider the regional status of COVID-19 extension and lockdown in the area of each hospital [1]. However, we recognize the practical difficulties in quantitatively measuring COVID-19 infection status in the exact area and period at each hospital. Instead, we believe that the data on COVID-19 events in the hospitals could practically represent the extension of COVID-19 in them.

Since outbreak clusters or staff infections seem to directly affect the medical resources for surgery or chemotherapy, we initially thought that outbreak clusters or infections in musculoskeletal tumor team staff could affect treatment status or strategy. Surprisingly, while strategies changed, the actual functional loss of the hospital was affected by infections in patients rather than cluster outbreaks or infections in staff (Figure 4). This suggests that hospitals were generally able to manage staff-related infections and outbreak clusters without significant disruption to services. In contrast, when patients were infected, institutions often postponed elective surgeries and chemotherapy, likely as a safety measure to minimize risk to patients undergoing treatment.

### 4.3. Limitations

This study has several limitations. First, clinical deterioration and care delays were based on subjective assessments reported by participating institutions. The absence of objective time-based or patient-level data introduces the potential for recall and reporting bias, which may affect the accuracy and consistency of the findings. To enhance objectivity and comparability, quantitative data should be incorporated in future investigations. Second, the study lacks a pre-pandemic control group, which limits our ability to directly attribute the observed disruptions to the COVID-19 pandemic. Without baseline data for comparison, we cannot exclude the possibility that some of the observed issues predated the pandemic. This limitation may be addressed by employing longitudinal study designs or retrospective controls in future research. Third, while internal comparisons among care components provided insight into perceived areas of impact, they do not allow conclusions regarding external influences or causality. These findings should be seen as exploratory, and further clarification of underlying factors should be pursued through studies incorporating objective external data, such as regional infection trends and institutional policies. Finally, although we compared results by hospital type (e.g., university hospitals, cancer centers), we did not conduct multivariable analyses to adjust for potential confounders such as hospital size or resource availability. In subsequent analyses, multivariable statistical approaches should be applied to control for institutional variability and improve interpretability. Given these limitations, the findings of this study should be interpreted with caution and considered as exploratory insights that highlight the need for more robust, data-driven investigations.

The most important clinical question regarding musculoskeletal tumor treatment during the COVID-19 pandemic is how the oncological outcomes changed due to the COVID-19 pandemic. To date, this question remains unanswered [3]. For example, prolongation of the time to treat intention for more than 30 days was reported to be unrelated to oncological outcomes, such as local relapse-free or disease-specific survival in soft tissue sarcomas, although metastasis-free survival did change [14]. In other studies, it has also been shown that the delay in the diagnosis of bone and soft tissue tumors itself does not have a statistically significant impact on overall survival [22]. Future studies should confirm the effects of related factors, such as delay or reduction in surgery, chemotherapy, or radiotherapy, as well as reduction in the referral of patients during the COVID-19 pandemic, on oncological outcomes.

## 5. Conclusions

During the COVID-19 pandemic, decreases in patient referrals, postponements and cancellations of surgical interventions, and prolongation of follow-up intervals were significant issues in managing musculoskeletal tumors at specialized hospitals in Japan. COVID-19 infections in patients, rather than cluster outbreaks in hospitals or infections in staff, impacted the treatment strategy for musculoskeletal tumors.

## Figures and Tables

**Table 1:** The questions on treatment status during the COVID-19 pandemic.

Q1	What is the Category of Your Hospital?
Q2	Did your hospital experience COVID-19 infections in patients undergoing treatment for bone/soft tissue tumors? If so, what was the status of patients’ treatment processes?
Q3	Did an outbreak cluster of COVID-19 occur in your hospital?
Q4	Did your department experience COVID-19 infections in the musculoskeletal team staff?
Q5	Did patient referrals change during the COVID-19 pandemic?
Q6	Did the hospital restrict the performance of radiological examinations (CT, MRI) during the COVID-19 pandemic?
Q7	Did the interval between the first visit and the performance of a biopsy change?
Q8	Did the performance of the following treatment process for primary malignant/intermediate bone and soft tissue tumors change during the COVID-19 pandemic?
	(A) The interval between the decision to apply and the day of chemotherapy.
	(B) The application frequency of chemotherapy.
	(C) The interval between the decision to apply and the day of surgery.
	(D) The application frequency of surgery.
	(E) The interval between the decision to apply and the day of radiotherapy.
	(F) The application frequency of radiotherapy.
Q9	Did the performance of the following treatment process for metastatic bone tumors change during the COVID-19 pandemic?
	(A) The interval between the decision to apply and the day of the surgery.
	(B) The application frequency of surgery.
	(C) The interval between the decision to apply and the day of radiotherapy.
	(D) The application frequency of radiotherapy.
Q10	Did the performance of palliative care change during the COVID-19 pandemic?
Q11	Was there any occasion to change the following aspects of the treatment strategy during the COVID-19 pandemic?
	(A) The cancellation or postponement of surgery that is usually given without any adjuvant therapy.
	(B) The addition of neoadjuvant chemotherapy/radiotherapy that is not usually given.
	(C) The cancellation of perioperative chemotherapy that is usually given.
	(D) The cancellation of the perioperative radiotherapy that is usually given.
	(E) The prolongation of the interval between the discharge and the first follow-up visit to the outpatient clinic.
	(F) The cancellation of chemotherapy for advanced cases that is usually given.
	(G) A change in the chemotherapy regimen for advanced cases that is usually given.

Note: COVID-19, coronavirus disease 2019; CT, computed tomography; MRI, magnetic resonance imaging.

**Table 2:** The impact of the COVID-19 pandemic on diagnostic processes.

		*n*	%
Q5	Patient referrals		
	No change	50	64.1
	Decrease	23	29.5
	Increase	5	6.4
Q6	Institutional restrictions on radiological examinations		
	No	73	93.6
	Yes	5	6.4
Q7	The interval between the first visit and the biopsy		
	No change	76	97.4
	Prolonged	2	2.6

**Table 3:** The impact of the COVID-19 pandemic on the treatment process for primary malignant bone and soft tissue tumors.

Q8		*n*	%
A	The interval between the decision to apply and the day of chemotherapy		
	No change	66	84.6
	Prolonged	5	6.4
	Not performed in the institution	7	8.9
B	The application frequency of chemotherapy		
	No change	62	79.5
	Decreased	6	7.7
	Increased	3	3.8
	Not performed at the hospital	7	8.9
C	The interval between the decision to apply and the day of surgery		
	No change	53	67.9
	Decreased	6	7.7
	Prolonged	18	23.1
	Not performed at the hospital	1	1.3
D	The application frequency of surgery		
	No change	50	64.1
	Decreased	23	29.5
	Increased	4	5.1
	Not performed at the hospital	1	1.3
E	The interval between the decision to apply and the day of radiotherapy		
	No change	69	88.5
	Decreased	3	3.8
	Prolonged	1	1.2
	Not performed in the institution	5	6.4
F	The application frequency of radiotherapy		
	No change	67	85.9
	Decreased	4	5.1
	Increased	2	2.5
	Not performed at the hospital	5	6.4

**Table 4:** The impact of the COVID-19 pandemic on the treatment process for metastatic bone tumors.

Q9		*n*	%
A	The interval between the decision to apply and the day of surgery		
	No change	68	87.1
	Decreased	2	2.6
	Prolonged	8	10.3
B	The application frequency of surgery		
	No change	60	77.0
	Decreased	13	16.7
	Increased	5	6.4
C	The interval between the decision to apply and the day of radiotherapy		
	No change	68	87.1
	Decreased	3	3.8
	Prolonged	2	2.6
	Not performed at the hospital	5	6.4
D	The application frequency of radiotherapy		
	No change	68	87.1
	Decreased	4	5.1
	Increased	1	1.3
	Not performed at the hospital	5	6.4

## Data Availability

The data collected through the survey were originally intended for presentation at a conference, and consent for public data sharing was not obtained from participants. Therefore, the data are not publicly available due to ethical and privacy considerations.
